# Two KTR Mannosyltransferases Are Responsible for the Biosynthesis of Cell Wall Mannans and Control Polarized Growth in *Aspergillus fumigatus*

**DOI:** 10.1128/mBio.02647-18

**Published:** 2019-02-12

**Authors:** Christine Henry, Jizhou Li, François Danion, Laura Alcazar-Fuoli, Emilia Mellado, Rémi Beau, Grégory Jouvion, Jean-Paul Latgé, Thierry Fontaine

**Affiliations:** a Aspergillus Unit, Institut Pasteur, Paris, France; b Necker-Enfants Malades Hospital, Department of Infectious Diseases and Tropical Medicine, Paris Descartes University, Paris, France; c Spanish Network for Research in Infectious Diseases (REIPI RD16/0016), ISCIII, Majadahonda, Madrid, Spain; d Unité de Neuropathologie Expérimentale, Institut Pasteur, Paris, France; Leibniz Institute for Natural Product Research and Infection Biology—Hans Knoell Institute Jena (HKI); Hans-Knoell-Institute; Stony Brook University; University of Texas MD Anderson Cancer Center

**Keywords:** *Aspergillus fumigatus*, galactomannan, KTR, mannosyltransferase, biosynthesis, cell wall

## Abstract

Fungal cell wall mannans are complex carbohydrate polysaccharides with different structures in yeasts and molds. In contrast to yeasts, their biosynthetic pathway has been poorly investigated in filamentous fungi. In Aspergillus fumigatus, the major mannan structure is a galactomannan that is cross-linked to the β-1,3-glucan-chitin cell wall core. This polymer is composed of a linear mannan with a repeating unit composed of four α1,6-linked and α1,2-linked mannoses with side chains of galactofuran. Despite its use as a biomarker to diagnose invasive aspergillosis, its biosynthesis and biological function were unknown. Here, we have investigated the function of three members of the Ktr (also named Kre2/Mnt1) family (Ktr1, Ktr4, and Ktr7) in A. fumigatus and show that two of them are required for the biosynthesis of galactomannan. In particular, we describe a newly discovered form of α-1,2-mannosyltransferase activity encoded by the *KTR4* gene. Biochemical analyses showed that deletion of the *KTR4* gene or the *KTR7* gene leads to the absence of cell wall galactomannan. In comparison to parental strains, the *Δktr4* and *Δktr7* mutants showed a severe growth phenotype with defects in polarized growth and in conidiation, marked alteration of the conidial viability, and reduced virulence in a mouse model of invasive aspergillosis. In yeast, the KTR proteins are involved in protein 0- and N-glycosylation. This study provided another confirmation that orthologous genes can code for proteins that have very different biological functions in yeasts and filamentous fungi. Moreover, in A. fumigatus, cell wall mannans are as important structurally as β-glucans and chitin.

## Abstract (precis)

The fungal cell wall is a complex and dynamic entity essential for the development of fungi. It allows fungal pathogens to survive environmental challenge posed by nutrient stress and host defenses, and it also is central to polarized growth. The cell wall is mainly composed of polysaccharides organized in a three-dimensional network. Aspergillus fumigatus produces a cell wall galactomannan whose biosynthetic pathway and biological functions remain poorly defined. Here, we described two new mannosyltransferases essential to the synthesis of the cell wall galactomannan. Their absence leads to a growth defect with misregulation of polarization and altered conidiation, with conidia which are bigger and more permeable than the conidia of the parental strain. This study showed that in spite of its low concentration in the cell wall, this polysaccharide is absolutely required for cell wall stability, for apical growth, and for the full virulence of A. fumigatus.

## INTRODUCTION

In fungi, mannans are composed of both short *O*- and *N*-linked mannans decorating glycoproteins and long mannan chains which are a major component of the fungal cell wall. *N*-linked and *O*-linked glycans have similar branched structures in yeasts and molds (1), and their synthesis follows the same pathway in yeasts and molds (2–6). In contrast, major differences have been found in the structural organization of the long mannans present in the cell wall of yeasts and filamentous fungi. In yeasts such as Saccharomyces cerevisiae and Candida albicans, highly branched *N*-linked mannans are bound to proteins and total more than 100 mannosyl residues per molecule. These mannans cover the surface of the cell wall and are not covalently bound to the polysaccharide core of the cell wall. In contrast, in Aspergillus fumigatus, which can serve of a model for ascomycetous molds, cell wall mannans (with an average of 80 mannose residues per chain) are integral parts of the cell wall since they are covalently bound to the glucan-chitin core (7). In addition, *Aspergillus* mannan contains galactofuranose residues and is released during fungal infection, making it an immunomodulatory molecule and a biomarker used to diagnose invasive aspergillosis (8–10). However, to date its biosynthesis had been unknown.

Pioneering studies in the model species S. cerevisiae have identified a series of mannosyltransferases which are sequentially involved in mannan biosynthesis. Addition of the first α1,6 mannose to the N-glycan of the glycoprotein is catalyzed by Och1p. Extension of the α-1,6-mannan chain is due to the multienzymatic complexes mannan polymerase I (Pol I) and Pol II (11). Proteins of these complexes are encoded by Mnn9, Van1, Anp1, Mnn10, Mnn11, and Hoc1 genes. Branching of the α-1,6-mannan backbone is initiated by Mnn2 and Mnn5 to produce short α-1,2-mannan oligosaccharides. This branched-core mannan is subsequently modified by phosphorylation by Mnn6 and can be capped by α-1,3-mannose added by Mnn1. Even though the genes involved in mannan synthesis have been identified, the respective synthetic events in the biosynthetic organization of yeast mannans are not fully understood.

In filamentous fungi and especially in Aspergillus fumigatus, the model species in which the cell wall has been the most extensively studied, synthesis of the cell wall-bound mannan has not been elucidated. The mannan of A. fumigatus which is bound to the β-1,3-glucan-chitin core of the cell wall is a linear polymer with a repeating unit composed of four α-1,6- and α-1,2-linked mannoses with side chains of galactofuran (7, 12). The *in silico* search for putative yeast mannosyltransferases in A. fumigatus has identified 11 orthologous yeast α-1,6- and α-1,2-mannosyltransferases. Accordingly, the deletion of all 11 of the putative orthologs of the yeast mannosyltransferases responsible for establishing α-1,6- and α-1,2-mannose linkages was undertaken in A. fumigatus (13). However, even though the encoded A. fumigatus proteins had an active mannosyltransferase activity which was able to complement the yeast genes (13, 14), the successive deletion of these 11 mannosyltransferase genes did not affect the mannan content of the mycelial cell wall of A. fumigatus. The only phenotype of this mutant was a reduction of the mannan content of the conidial cell wall leading to a partial disorganization of the cell wall and defects in conidial dormancy without affecting the structural cell wall galactomannan (GM) of the mycelium. That study suggested that at least two sets of mannosyltransferases are responsible for the synthesis of cell wall mannans in the conidium and mycelium of A. fumigatus. Moreover, that study showed that genes with highly conserved sequence can code for proteins which have very different biological functions in yeasts and filamentous fungi. These considerations led us to investigate other orthologs of yeast mannosyltransferases with a function not associated with mannan polymerization in yeast. This is why we investigated the *KTR* genes, which were excluded from our initial study (13) for two reasons: (i) in yeast, these genes are exclusively involved in N- and O-glycan processing in yeast (15); (ii) the gene coding for one member of this family which contains three genes has been previously disrupted without any significant effect on the growth phenotype of the mutant (4). The data of this current study showed that in A. fumigatus the two members of the KTR family orthologous to the yeast *KTR4* and *KTR7* are mannosyltransferases responsible for the polymerization of the structural cell wall galactomannan. Their deletion led to a severe growth phenotype with a hyperbranched mycelium resulting from a lowered polarized growth, a strong defect in conidiation, a marked alteration of the conidial viability over time, and a reduction of virulence in mouse models.

(This work was presented in part at the Fungal Cell Wall 2017 Conference [Ensenada, Baja California, Mexico, 9 to 12 October 2017) and at the 4th Glycobiology World Congress (Rome, Italy, 17 to 18 September 2018.)

## RESULTS

### Phylogenetical analysis of Ktr proteins in A. fumigatus and construction of ΔKtr mutant and revertant strains.

**FIG 1:**
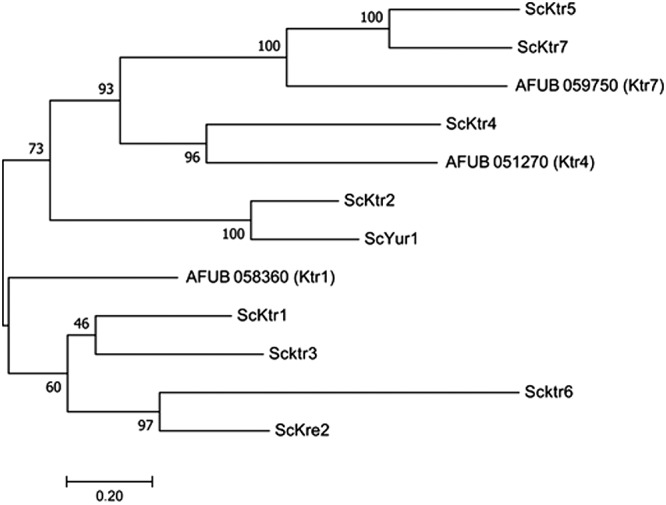
Phylogenetical tree of KTR genes in A. fumigatus and S. cerevisiae.

The A. fumigatus *KTR* genes have very close sequence homologies with the S. cerevisiae KTR genes which belong to the GT15 family (www.Cazy.org). A phylogenetical analysis performed with the MUSCLE software with the 9 KTR members of S. cerevisiae showed that A. fumigatus UB_059750 (AFUA_5G12160) and AFUB_051270 (AFUA_5G02740) genes were closely related to ScKtr7 and ScKtr4, respectively (Fig. 1). The third member (AFUB_058360 [AFUA_5G10760]) was more distantly related to the yeast Ktr members (Fig. 1). In accordance with this phylogenetic tree, AFUB_058360, AFUB_051270, and AFUB_059750 were named KTR1, KTR4, and KTR7, respectively. These three proteins share 31% to 37% identity. Transcriptome sequencing (RNAseq) gene expression data from A. fumigatus have been recently published (16). All three KTR genes are expressed in resting conidia, germ tubes, and mycelia. In order to investigate the function of these KTR genes in A. fumigatus, deletion of *KTR1*, *KTR4*, and *KTR7* and construction of the complemented strains were carried out with the β-rec/six system as described earlier (17). The validation of mutant strains was done by Southern blot analysis as shown in Fig. S1 in the supplemental material.

10.1128/mBio.02647-18.1 FIG S1 Southern blots of *Δktr1* (A), *Δktr4* (B), and *Δktr7* (C) mutants and *Δktr4*::*KTR4* and *Δktr7*::*KTR7* revertant strains and targeted replacement strategies used for A. fumigatus *KTR* genes. Each panel shows the restriction maps of the *KTR* deletion constructs after the integration of the βrec/hygromycin resistance marker at the *KTR* locus and the Southern blot analysis of the parental strain, i.e., the *ktr* mutant strain. Genomic DNA of each strain was digested with appropriate restriction enzymes and hybridized with a specific probe. Revertant strains *Δktr4*::*KTR4* and *Δktr7*::*KTR7* were obtained after excision of the βrec/hygromycin marker followed by reintroduction of the respective parental genes using the reusable βrec/hygromycin cassette. Download FIG S1, PDF file, 0.2 MB. Copyright © 2019 Henry et al. 2019 Henry et al. This content is distributed under the terms of the Creative Commons Attribution 4.0 International license.

### Mycelial growth and morphology.

**FIG 2:**
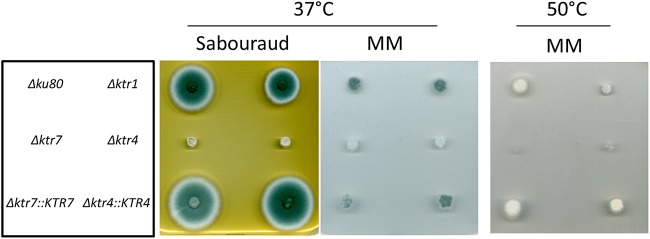
Growth of the parental *Δku80* strain, *Δktr1*, *Δktr4* and *Δktr7* mutants, and *Δktr4*::*KTR4* and *Δktr7*::*KTR7* revertant strains on solid media. Strains were grown on either Sabouraud or minimum (MM) solid medium. A total of 10³ conidia of each strain were spotted on media and incubated at 37°C or 50°C for 48 h.

**FIG 3:**
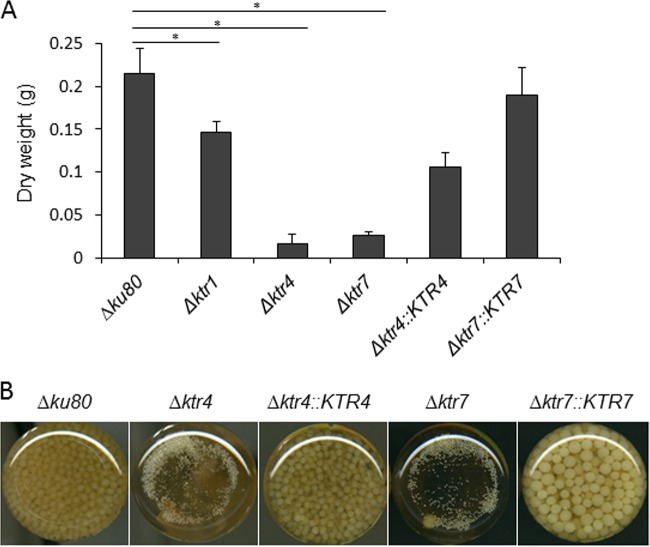
Growth of parental *Δku80* strain, *Δktr1*, *Δktr4*, and *Δktr7* mutants, and *Δktr4*::*KTR4* and *Δktr7*::*KTR7* revertant strains in liquid media. Strains were grown in Sabouraud liquid media. Flasks (50 ml) were inoculated with 10^6^ conidia and incubated under shaking conditions (150 rpm/min) at 37°C for 24 h. (A) Mycelium biomass. The biomass/flask was quantified by the weight of the mycelium after drying at 80°C. Each value of mycelium dry weight represents the average of data from three independent replicates (*, *P* < 0.05; error bars represent standard deviations). (B) Gross morphology of mycelium.

**FIG 4:**
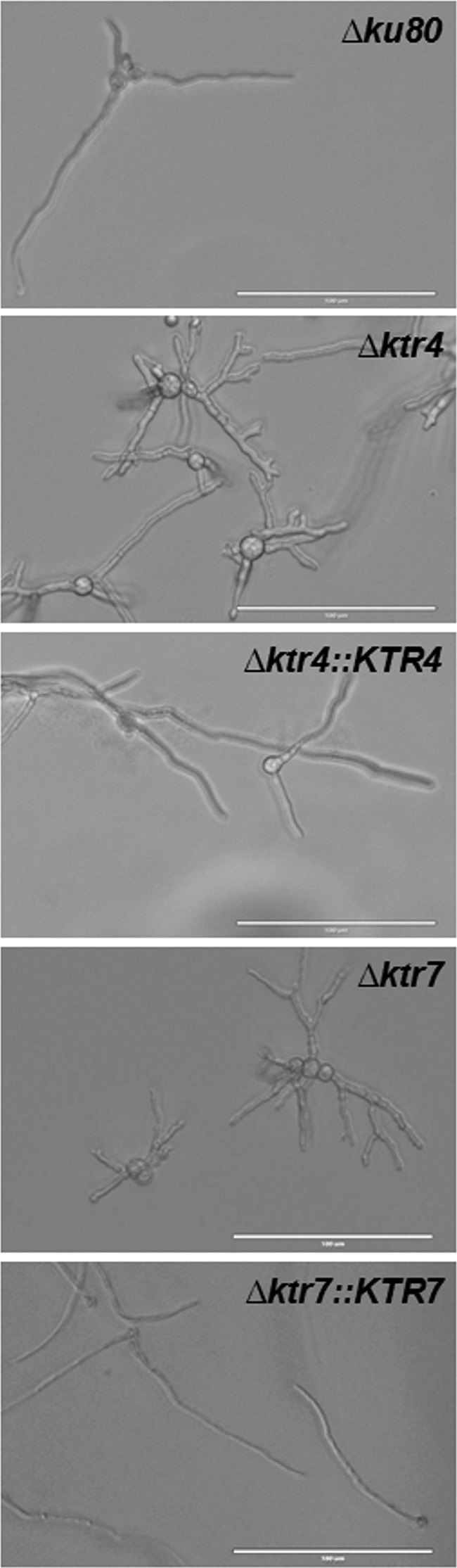
Morphology of germinated conidia of parental *Δku80* strain, *Δktr4* and *Δktr7* mutants, and Δ*ktr4*::*KTR4* and *Δktr7*::*KTR7* revertant strains after 20 ± 2 h of incubation in MM liquid medium at 37°C. White bars represent 100 µm.

Parental, *Δktr*, and complemented strains were grown in liquid and on agar minimum media (MM) and Sabouraud media. After 2 days at 37°C, the growth of *Δktr4* and *Δktr7* mutants was severely reduced in MM as well as on Sabouraud agar media (Fig. 2). The growth defect was more severe at 50°C, under which conditions almost no growth of *Δktr4* and *Δktr7* mutants was observed. In contrast, the *Δktr1* mutant and *Δktr4*::*KTR4* and *Δktr7*::*KTR7* complemented strains showed normal growth comparable to that seen with the parental strain. The addition of 6% KCl or 1 M sorbitol to the medium as an osmostabilizer did not restore the parental growth phenotype (data not shown). A statistically significant reduction of growth of *Δktr4* and *Δktr7* mutants was also observed in liquid media (Fig. 3). After 18 to 22 h of culture in liquid media, parental and complemented strains produced long and thin hyphae (Fig. 4). In contrast, hyphae of *Δktr4* and *Δktr7* mutants were denser and hyperbranched. Accordingly, *Δktr4* and *Δktr7* mutants produced numerous small mycelium balls in both versions of the liquid media (Fig. 3B).

### Sensitivity to drugs.

Both the *Δktr4* and *Δktr7* mutants were more sensitive to calcofluor white (CFW), Congo red, and SDS than the parental strain and the *Δktr1* mutant and their respective complemented strains (see Table S1 in the supplemental material). MICs of 7.5, 2.5, and 100 µg/ml were estimated for calcofluor white, Congo red, and SDS, respectively, for both the *Δktr4* and *Δktr7* mutants versus MICs of 30 and 40 µg/ml and 1 mg/ml, respectively, for the parental strain, suggesting an alteration of the cell wall organization or permeability. In contrast, no difference of growth was observed between the parental and all mutant strains in the presence of menadione, H_2_O_2_, amphotericin B, caspofungin, posaconazole, or itraconazole (Table S1).

10.1128/mBio.02647-18.7 TABLE S1 MIC of antifungal drugs. Download Table S1, DOCX file, 0.03 MB. Copyright © 2019 Henry et al. 2019 Henry et al. This content is distributed under the terms of the Creative Commons Attribution 4.0 International license.

### Conidiation, conidial morphology, conidium survival, and conidial germination.

In comparison to the parental strain, the *Δktr1* mutant, and the *Δktr4*::*KTR4* and *Δktr7*::*KTR7* complemented strains, the *Δktr4* and *Δktr7* mutants produced very low amounts of conidia, representing 1.4% and 4.6% of the total amount of conidia produced by the parental strain in malt medium, respectively, and 4.4% and 10.2% in malt supplemented with 6% KCl, respectively (Table 1). The supplementation of the medium with 6% KCl or 1 M sorbitol (data not shown) did not restore conidiation, showing that the strong conidiation defect of both the *Δktr4* and *Δktr7* mutants was not due to higher sensitivity to osmotic pressure of the mutants. The morphology of the conidia of the *Δktr4* and *Δktr7* mutants was altered. When they were produced on malt-KCl medium, conidia from the parental and complemented strains and the *Δktr1* mutant appeared as round spheres with an average diameter of 3 (±0.4) µm. Conidia produced by the *Δktr4* and *Δktr7* mutants were heterogeneous in size and larger than those produced by the parental strain, with average diameters of 3.4 µm (±0.9 µm) and 4.05 µm (±1.5 µm), respectively. In addition, the level of labeling of the *Δktr4* and *Δktr7* mutant conidia by CFW was much higher than that observed with the parental strain. Moreover, the levels of labeling of the conidia with fluorescein isothiocyanate (FITC) were also different. The conidia from parental and complemented strains were FITC labeled (at the cell wall) to only a slight extent, whereas 66% and 31% of *Δktr4* and *Δktr7* mutant conidia, respectively, were strongly intracellularly labeled (Fig. S2), suggesting that the deletion of *KTR4* and *KTR7* induced an increase of conidial permeability. This defect in cell wall permeability was associated with a reduction of conidia viability (Fig. S3). For example, conidia from the parental and complemented strains were resistant to storage in water at 4°C whereas 59% and 93% of *Δktr4* mutant conidia died after 1 and 2 months of storage under the same condition. Similar loss of viability was obtained for the *Δktr7* mutant (data not shown).

**TABLE 1:** Conidiation of the *Δktr* mutants and the parental *Δku80* strains on malt agar in the presence or absence of 6% KCl^*a*^

Strain	Growth condition	
	Malt	Malt–6% KCl
Δ*ku80*	1,865 ± 688 × 10^6^	1,199 ± 205 × 10^6^
Δ*ktr1*	2,663 ± 338 × 10^6^	ND
Δ*ktr4*	26.8 ± 12.5 × 10^6^	55 ± 10 × 10^6^
Δ*ktr7*	82.3 ± 24 × 10^6^	122 ± 33 × 10^6^
Δ*ktr4*::KTR4	1,714 ± 305 × 10^6^	878 ± 123 × 10^6^
Δ*ktr7*::KTR7	1,711 ± 475 × 10^6^	727 ± 89 × 10^6^

a Strains were inoculated by spanning 50 µl of suspensions of 10^6^ conidia/ml on slants of malt or malt–6% KCl solid media and were incubated at 37°C for 1 night and 2 weeks at room temperature. Produced conidia were collected with 0.05% Tween 20 solution and quantified. Values represent means ± standard deviations (SD) of results from three different experiments. ND, not determined.

10.1128/mBio.02647-18.2 FIG S2 Conidial morphology of the parental *Δku80* strain, the *Δktr4* and *Δktr7* mutants, and the *Δktr4*::*KTR4* and *Δktr7*::*KTR7* revertant strains. Conidia were collected after three weeks of growth on malt–6% KCl solid medium at room temperature. Conidia were stained with either FITC (1 mg/ml) or calcofluor white (0.5 µg/ml) and observed under a fluorescence microscope. White bars represent 10 µm. Download FIG S2, PDF file, 0.9 MB. Copyright © 2019 Henry et al. 2019 Henry et al. This content is distributed under the terms of the Creative Commons Attribution 4.0 International license.

10.1128/mBio.02647-18.3 FIG S3 Conidial viability of the parental *Δku80* strain, the *Δktr4* mutant, and the *Δktr4*::*KTR4* revertant strain. Conidial viability was estimated after the storage of 10³ conidia/ml 0.05% Tween 20 solution at 4°C for 1 and 2 months. The percentage of survival was estimated by CFU quantification after spreading on 2% malt agar plates. Each value represents the average of data from three independent replicates (error bars represent standard deviations). Download FIG S3, PDF file, 0.05 MB. Copyright © 2019 Henry et al. 2019 Henry et al. This content is distributed under the terms of the Creative Commons Attribution 4.0 International license.

**FIG 5:**
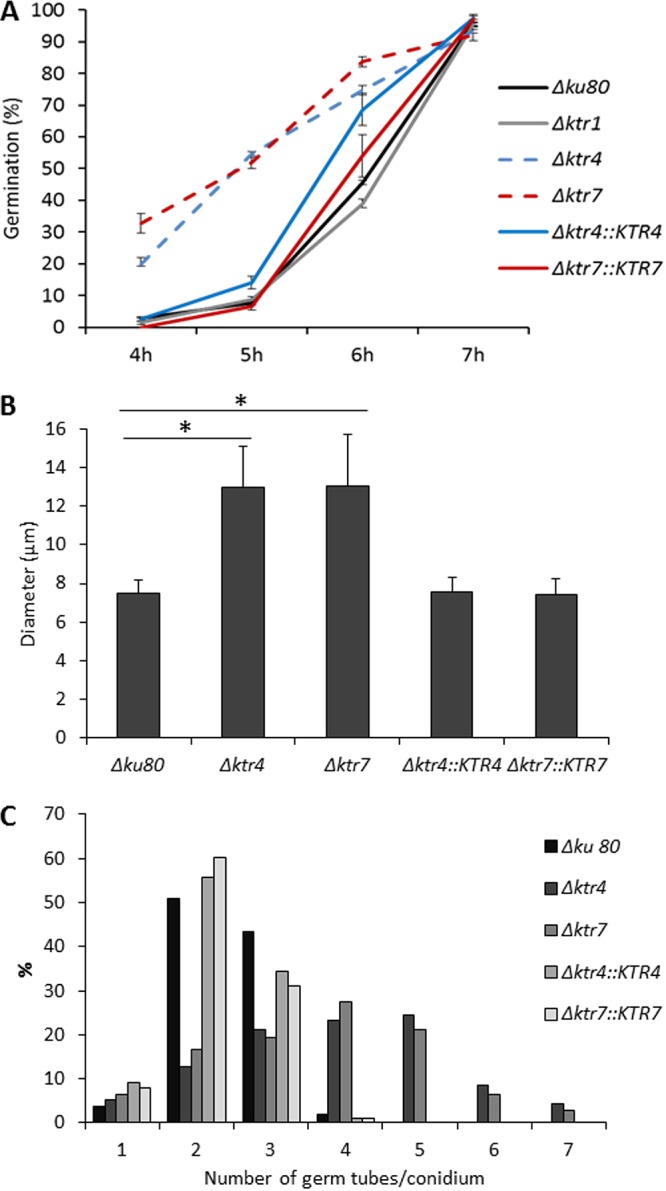
Conidial germination of parental *Δku80* strain, *Δktr1*, *Δktr4*, and *Δktr7* mutants, and Δ*ktr4*::*KTR4* and *Δktr7*::*KTR7* revertant strains. (A) Germination. Conidia (5 µl of 2.10^6^ conidia/ml suspension) were spotted on a slide of Sabouraud agar medium and incubated at 37°C under conditions of a humid atmosphere. Percentage of germination was quantified by bright-field counting of germinated and nongerminated conidia under a microscope. Each value represents the average of data from three independent replicates. Differences in the percentages of germination of *Δktr4* and *Δktr7* mutants were significant at 4, 5, and 6 h (*P* < 0.0001; error bars represent standard deviations). (B and C) The size of germinated conidia (B) and the number of germ tubes/conidium (C) were determined after 16 h of incubation in liquid MM medium at 37°C. Each value represents an average of data resulting from the counting of hundred conidia. (*, *P* < 0.001; error bars represent standard deviations).

The *Δktr4* and *Δktr7* mutants germinated faster than their parental strain. On malt agar, conidia from parental, *Δktr1* mutant, and *Δktr4*::*KTR4* and *Δktr7*::*KTR7* complemented strains started to germinate after 4 h of incubation in the medium whereas 20% and 32% of *Δktr4* and *Δktr7* conidia, respectively, had already germinated by the same time (Fig. 5A). After 5 h, 50% of *Δktr4* and *Δktr7* conidia had germinated versus 24% of the conidia of the parental strain. Moreover, the pattern of germination of the *Δktr4* and *Δktr7* mutant strains was different from that seen with the parental strain. First, swollen conidia from *Δktr4* and *Δktr7* mutant strains were 1.7 times larger and more heterogeneous in size than those from parental strain (Fig. 5B). Second, the emergence of germ tubes was different in the parental and mutant strains. Germinating conidia of the parental strain produced successively first, second, and third germ tubes (see Movie S1 in the supplemental material). At 16 h, 51% and 41% of the parental conidia produced 2 and 3 germ tubes, respectively, with an average of 2.4 germ tubes/conidium (Fig. 5C). The second germ tube was issued at 180° from the first one; the third one at 90°. In contrast, the conidia of the *Δktr4* and *Δktr7* mutant strains produced up to 7 germ tubes, with averages of 3.7 and 3.9 germ tubes/conidium, respectively (Fig. 5C; see also Movie S2), and several germ tubes emerged simultaneously from swollen *Δktr* conidia without any specific location of the emergence site. Taken together, these data showed that the deletion of *KTR4* and *KTR7* affected growth polarization during germ tube emergence.

10.1128/mBio.02647-18.9 MOVIE S1 Germination of conidia of the parental *Δku80* strain. Download Movie S1, MOV file, 2.5 MB. Copyright © 2019 Henry et al. 2019 Henry et al. This content is distributed under the terms of the Creative Commons Attribution 4.0 International license.

10.1128/mBio.02647-18.10 MOVIE S2 Germination of conidia of the *Δktr4* mutant strain. Download Movie S2, MOV file, 2.7 MB. Copyright © 2019 Henry et al. 2019 Henry et al. This content is distributed under the terms of the Creative Commons Attribution 4.0 International license.

### Protein glycosylation and cell wall analysis.

**FIG 6:**
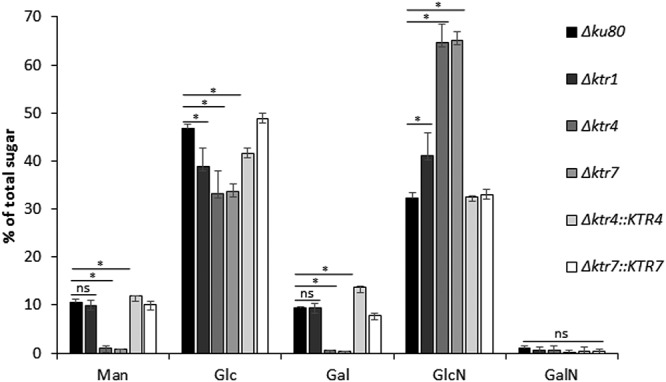
Monosaccharide composition of the alkali-insoluble fraction of the cell wall of the parental *Δku80* strain, *Δktr1*, *Δktr4*, and *Δktr7* mutants, and Δ*ktr4*::*KTR4* and *Δktr7*::*KTR7* revertant strains. The cell wall alkali-insoluble fraction (AI) was purified from mycelium grown for 24 h in liquid Sabouraud medium at 37°C (expressed as a percentage of total hexoses plus hexosamines). Monosaccharides were quantified after total acid hydrolysis and chromatography analysis. Each value represents an average of data from three independent triplicates. Statistical differences between parental and mutant strains are indicated as follows: ns, not significant; *, *p* < 0.01; error bars represent standard deviations. Man, mannose; Glc, glucose; Gal, galactose; GlcNAc, N-acetylglucosamine; GalNAc, N-acetylgalactosamine.

Matrix-assisted laser desorption ionization–time of flight (MALDI-TOF) spectra of protein N-glycans were similar for the parental and *Δktr4* and *Δktr7* mutant strains, with ion masses from *m*/*z* 1,419.55 to 2,391.91 at an increment of 162 (Fig. S4). These ion masses were in agreement with the presence of 2 GlcNAc and 6 to 12 hexose residues as previously described (18). These data indicated that the deletion of *KTR4* and *KTR7* did not induce a modification of the N-glycosylation of proteins. In contrast, the mannan content of the cell wall was affected by the deletion of *KTR4* and *KTR7*. The chemical composition of the alkali-insoluble (AI) and alkali-soluble (AS) fractions of the A. fumigatus cell wall of parental and mutant strains has been analyzed. No significant difference was observed in the monosaccharide content of the AS fraction of the *Δktr4* and *Δktr7* mutant strains in comparison to the parental and complemented strains (data not shown). On the other hand, an almost complete loss of the galactomannan content associated with a compensatory doubling of the chitin amount was observed in the AI fraction of the *Δktr4* and *Δktr7* mutants in comparison to the parental strain (Fig. 6). These data showed that deletion of *KTR4* and *KTR7* led to the loss of the cell wall galactomannan (GM) cross-linked to the glucan-chitin core and suggested that Ktr4p and Ktr7p were mannosyltransferases involved in the biosynthesis of the GM.

10.1128/mBio.02647-18.4 FIG S4 MALDI-TOF mass spectra of N-glycans. MALDI-TOF mass spectra of N-glycans purified from secreted proteins produced by the parental *Δku80* strain (A), the *Δktr4* mutant (B), and the *Δktr7* (C) mutant in liquid Sabouraud medium are shown. Mass spectra were acquired using FlexControl software, and shots were recorded in positive reflectron mode. Ion mass (*m*/*z*) data correspond to [M + Na]^+^ (HexNac, N-acetylhexosamine; Hex, hexose). Download FIG S4, PDF file, 0.1 MB. Copyright © 2019 Henry et al. 2019 Henry et al. This content is distributed under the terms of the Creative Commons Attribution 4.0 International license.

### Mannosyltransferase activity of the recombinant protein Ktr4p.

**FIG 7:**
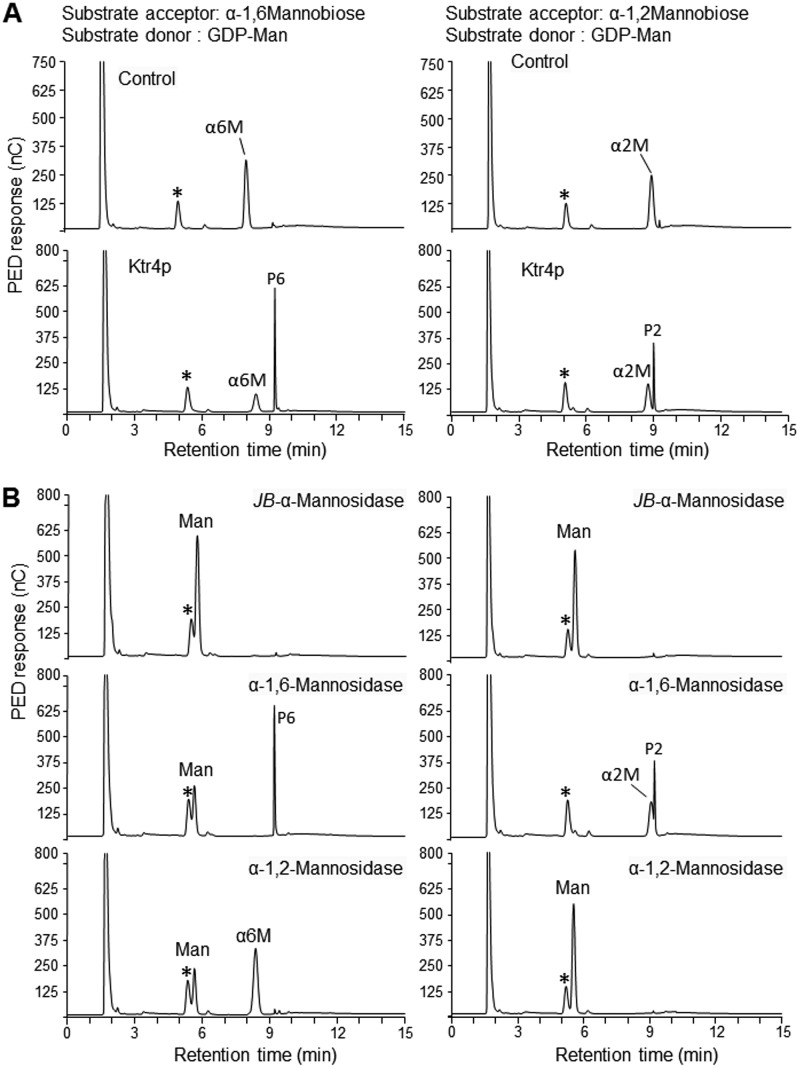
Ktr4p enzyme activity. (A) HPLC analysis of the products obtained after incubation of recombinant ktr4p with GDP-mannose and mannobiose (α-1,6-mannobiose or α-1,2-mannobiose). HPLC profiles were obtained after incubation of nontreated (Ktr4p) or heat-inactivated (Control) Ktr4p with mannobiose and GDP-mannose. (B) HPLC analysis of products submitted to exo-α-mannosidase digestions (PED, pulsed electrochemical detection; *JB*-α-Mannanase, *Jack bean* α-mannosidase; α2M, α-1,2-mannobiose; α6M, α-1,6-mannobiose; Man, mannose; *, contaminant from buffer; P6, trisaccharide obtained in the presence of α-1,6-mannobiose; P2, trisaccharide obtained in the presence of α-1,2-mannobiose). *Jack bean* α-mannosidase fully degraded both acceptors (α2M and α6M) and products (P2 and P6) to release mannose residue, showing the α configuration of all mannose residues. α-1,6-Mannosidase degraded the α6M but not the P6. α-1,2-Mannosidase degraded α2M and P2 into mannose and P6 into α6M and mannose.

A Ktr4p recombinant (r-Ktr4) protein with a *M*_r_ of 43 kDa was produced in Escherichia coli. To test the putative mannosyltransferase activity, the r-Ktr4 protein was incubated with GDP-mannose as the substrate donor and α-1,2 mannobiose or α-1,6 mannobiose as the acceptor. Ktr4p was able to use both acceptors to produce a new product (Fig. 7). Gel filtration chromatography performed on a TSK-G-oligo-PW column and MALDI-TOF mass spectrometry have shown that the products obtained with an α-1,2 mannobiose or α-1,6 mannobiose acceptor were trisaccharides. The trisaccharide products were then characterized by exo-α-mannosidase digestion. For both acceptors, the reaction products were resistant to α-1,6-mannosidase treatment but were degraded by jack bean α-mannosidase and α-1,2-mannosidase treatments (Fig. 7), showing that the trisaccharide products contained a mannose at the nonreducing end linked through a α-1,2 linkage. These data showed that Ktr4p had α-1,2-mannosyltransferase activity and was able to use both α-1,2- and α-1,6-mannobiose as an acceptor to add one mannose residue.

### Virulence.

The virulence of the *Δktr4* mutant was tested in a mouse model of invasive aspergillosis using cyclophosphamide/cortisone acetate as an immunosuppressive treatment. The parental and complemented strains showed the same survival curves, with no mouse surviving 6 days after infection, whereas 30% of the mice remained alive in the cohort infected by the *Δktr4* mutant. Statistical analysis showed that the *Δktr4* mutant was significantly less virulent than the parental and complemented strains (Fig. S5). Histopathological analysis was performed 3 days postinfection. Multifocal inflammatory lesions, centered on bronchi/bronchioles and with secondary extension to alveoli, containing filamentous fungi were observed with both the parental and *Δktr4* strains (Fig. S6). However, the *Δktr4* mutant displayed reduced invasion of alveoli and large blood vessels in comparison to the parental strain.

10.1128/mBio.02647-18.5 FIG S5 Virulence of the parental *Δku80*, *Δktr4* mutant, and *Δktr4*::*KTR4* revertant strains in an invasive aspergillosis mouse model. Download FIG S5, PDF file, 0.04 MB. Copyright © 2019 Henry et al. 2019 Henry et al. This content is distributed under the terms of the Creative Commons Attribution 4.0 International license.

10.1128/mBio.02647-18.6 FIG S6 Histopathological sections of mouse lungs infected with A. fumigatus strains. (A to C) Parental strain (*Δku80*). (D to F) *Δktr4* mutant. (A and D, HE staining; B, C, E, and F, Gomori Grocott’s staining). (A to C) Lung section with *Δku80* strain. A multifocal inflammatory lesion, centered on bronchi/bronchioles (BB), with secondary extension to alveoli (black arrows), containing filamentous fungi (black arrowheads) is shown. (A and B) Fungi were located in the bronchi/bronchioles, with invasion of alveoli (black arrowheads) (B) and large blood vessels (BV). (C) Lung section with *Δktr4* mutant. (D to F) Same inflammatory lesion as shown for *Δku80* (D and E), with less invasion of alveoli by the fungus (black arrowheads) (E) and no invasion of large blood vessels (F). Download FIG S6, PDF file, 0.4 MB. Copyright © 2019 Henry et al. 2019 Henry et al. This content is distributed under the terms of the Creative Commons Attribution 4.0 International license.

## DISCUSSION

This study and previous ones have shown that a high number of mannosyltransferases are functional in A. fumigatus (4, 13, 14, 19). A major impediment to understanding the respective roles of all mannosyltransferases is their putative redundancy due to the lack of specificity of the different mannosyltransferases as seen at least *in vitro* in yeast, where mannosyltransferases can compensate for each other (7, 8). In S. cerevisiae, Ktr orthologs belong to the GT15 family in the CAZy database and have been characterized as α-1,2-mannosyltransferases playing a role in N- and O-mannosylation of glycoproteins (15, 20, 41, 53). Here, it was shown that although A. fumigatus ktr4p (Afktr4p) and Afktr7p share high sequence homologies with the yeast Ktrp orthologs and a mannosyltransferase activity, the deletion of A. fumigatus *KTR4* and *KTR7* genes did not alter protein mannosylation but led to the loss of the cell wall galactomannan. This report provides another clear example of the finding that orthologous genes code for proteins that may have very different biological functions in yeasts and filamentous fungi even though they have very related enzymatic activities, at least *in vitro*. It shows that the biosynthesis of the A. fumigatus mannans uses transferases and pathways different from those existing in yeasts. However, even in yeasts, the composition of the cell wall mannans, the number of genes involved in their synthesis, and the phenotype of the respective mutants differ from species to species. The elongation of the poly-α1,6-mannan backbone in S. cerevisiae is carried out by the mannose Pol I (Man Pol I) and Pol II complexes to build up the α-1,6-mannan backbone (21–24). Mnn9 is a member of these two complexes, but its quantitative contribution to α-1,6-mannan synthesis has not been clarified. In C. albicans, Mnn9 is the major contributor to the extension of the α-1,6-backbone since a deletion of *MNN9* resulted in the loss of 50% of the cell wall mannan. Another example can be found in the elongation of the α-1,2-mannan side chains. In S. cerevisiae, only 2 genes are responsible for the elongation of the α-1,2-mannan side chain whereas six paralogs of the Mnn2/Mnn5 genes were identified in C. albicans (54). We have previously shown that in A. fumigatus, genes orthologous to the yeast Man Pol I and II complexes are involved only in the synthesis of a specific amorphous alkali-soluble conidial mannan and are not involved in the synthesis of the main galactomannan constitutive of the cell wall of the mycelium and conidium (13, 14). This can be associated with the difference in the structures of long mannans in yeast and molds. In yeast, it is a branched structure directly anchored to proteins but not covalently bound to the cell wall structural polysaccharides, whereas in A. fumigatus, it is a repeat unit containing a linear structure covalently bound to the core polysaccharide.

*KTR1* is the third *KTR* homolog in A. fumigatus and has been previously analyzed by Wagener et al. (4). Deletion of the KTR1 gene was repeated in this study using the *akuB Δku80* CEA17 background, which was different from the wild-type strain D141 and its Δ*akuA* AfS35 derivative used previously by Wagener et al. (4) since different morphotypes from different genetic backgrounds can arise in A. fumigatus. The deletion of the KTR1 in the *akuB Δku80* CEA17 mutant confirmed the results from the previous study (4) and showed that deletion of *KTR1* led to a very weak phenotype compared to those seen with the *Δktr4* and *Δktr7* mutants. Similar findings were obtained in Beauveria bassiana, where deletion of *KTR4* and *KRE2* induced drastic phenotypes and deletion of *KTR1* led only to a decrease in fungal thermotolerance (25). Such a lack of functional redundancy between members of the same gene family is common in filamentous fungi and especially in A. fumigatus. Even in cases in which similar enzymatic functions have been identified in the different members of a family, the phenotypes of the mutant lacking the encoding gene can be very different, ranging from the absence of the corresponding phenotype to a drastic or even lethal phenotype. This has been shown for the members of the Gel family and the Chs family in A. fumigatus (26–28).

The two major morphological phenotypes of the *Δktr4* and *Δktr7* mutants are the production of poorly viable conidia and a strong defect in vegetative growth. The deletion of *KTR4* and *KTR7* induced a loss of polarity during conidial germination and hyphal elongation. Establishment of the polarity in filamentous fungi is composed of three sequential steps. First, there is a deposition of cortical markers which establishes an axis of polarity and deposit. Next, products of key polarity genes such as the *CDC42*/*RAC1* module and polarisome components are recruited to the cortical markers. Finally, activity of the cell growth machinery involving actin and tubulin and leading to the sequential supply of proteins, lipids, and polysaccharides to the hyphal tip deposition of cell wall biosynthetic enzymes adds new cellular components in the correct place, resulting in asymmetric growth (29–32). In addition to mannan synthesis, polarization of germ tube growth requires also an appropriate deposition of two other structural components of the cell wall (β-1,3-glucan and chitin) (26, 33). In contrast, the absence of synthesis of α-glucan or β-1,3/1,4-glucan or galactosaminogalactan (GAG) did not alter the filamentous polarized growth (34–36), showing that only the three-dimensional (3D) structural skeleton of the cell wall chitin-β-1,3-glucan-GM plays an essential role in the establishment of polarity. Interestingly, the absence of cell wall GM was not compensated by the presence of the high amount of chitin seen in the cell wall of the *Δktr4* and *Δktr7* mutants to maintain normal filamentous growth. The data from the current study provide a great example of the need to have balanced accumulations of structural cell wall polysaccharides to avoid a loss of the dominance of the apical Spitzenkörper in growing hyphae and for the maintenance of the right polarity. However, the data from this study show also that the requirement of cell wall organization is less important in liquid medium than under aerobic conditions, under which the filamentous growth is more severely altered. Similarly, in our experimental murine model of aspergillosis, the *Δktr4* mutant displayed reduced mycelium development which remained less pronounced than that seen in agar media (see Fig. S6 in the supplemental material), showing that the phenotype is highly dependent on growth conditions and explaining why the virulence defect was not so drastic as would be expected from the very altered *in vitro* phenotype.

This study showed that the GM cell wall biosynthesis pathway is independent of the protein mannosylation pathway and that ktr4 and ktr7 are key enzymes essential for the production of the constitutive cell wall galactomannan. Double deletion of KTR4 and KTR7 led to a phenotype similar to that seen with the single mutants (not shown), indicating that ktr4p and ktr7p are involved in the cell wall galactomannan biosynthetic pathway. The biosynthesis of galactomannan requires two different and separate pathways responsible for the synthesis of the galactofuran and the mannan elongation. First, the synthesis of the side chains of the GM is under the control of Golgi galactofuranosyltransferases, which use UDP galactofuranose as substrate (37, 38). However, the addition of galactofuranose is not a prerequisite either for the polymerization of mannan or for its incorporation in cell wall (39). Second, the mannan backbone assembly of galactomannan utilizes GDP-Man, which is transported at the Golgi level (40) and then used by Ktr proteins of A. fumigatus. These mannosyltransferases can accommodate either α-1,2-mannobiose or α-1,6-mannobiose as an acceptor. Since Ktr proteins in yeasts have described previously as Golgi resident α-1,2mannosyltransferases (4, 15, 41), these observations support a biosynthetic model according to which galactomannan is assembled in the Golgi apparatus and secreted to the plasma membrane before being cross-linked to cell wall β-glucan by extracellular transglycosidases. The chemical structure of the mannan chain (i.e., a tetra-α-1,2-mannoside repeat unit linked through an α-1,6 linkage) and its synthesis suggest specific forms of regulation of and/or cooperation between glycosyltransferases. Our current hypothesis is that Ktr4p and Ktr7p are α-1,2-mannosyltransferases acting sequentially during the synthesis of the tetra-α-1,2-mannoside unit. Examples of sequential addition of mannose residue have been described in yeast. Mnn2 and Mnn5 add the first and second branched α-1,2-mannose residues, respectively, on the α-1,6-mannan in yeast (42). The Bmt3 and Bmt1 β-1,2-mannosyltransferases add the first and second β-1,2-mannose residues, respectively, on N-mannan in C. albicans (43). These examples clearly show that the product of the first transferase activity is the acceptor of the second one and that the recognition of the acceptor by the transferase activity represents a critical point to drive the sequential addition of mannose residue. Such sequential activities could explain why both the *KTR4* and the *KTR7* genes are essential to the GM synthesis but are not redundant. The synthesis of the GM may also require the involvement of other α-1,2 and/or α-1,6-mannosyltransferase activities that remain to be identified to decipher the GM synthesis in A. fumigatus.

A recent study suggested that the DFG (for defective in filamentous growth) orthologs of the yeast DFG5/DCW1 are required for the cross-linking between GM and β-glucan (L. Muszkieta, T. Fontaine, R. Beau, I. Mouyna, M. S. Vogt, J. Trow, B. P. Cormack, L.-O. Essen, G. Jouvion, and J.-P. Latgé, submitted for publication). Interestingly, deletions of orthologous DFG genes in A. fumigatus led to phenotypes closely related to those seen with the *Δktr4* and *Δktr7* mutants, showing the importance of the cross-linked GM in the cell wall. The search for new transglycosidases or regulators and protein chaperones involved in the synthesis of the mannan chain is currently under way.

## MATERIALS AND METHODS

### Growth conditions.

The A. fumigatus parental strain used in this study was strain akuB *Δku80* pyrG^+^ (44). Conidia were produced on 2% malt agar slants after 2 weeks of growth at room temperature and were recovered by vortex mixing with 0.05% (vol/vol) Tween 20 aqueous solution. The fungus was grown on agar (2%) or in liquid culture media as follows: *Aspergillus* minimum medium (MM) (45), Sabouraud (2% glucose, 1% mycopeptone) (Difco), or 2% malt (Cristomalt). For production of conidia, mutant strains were grown on malt agar medium supplemented with 6% KCl.

### Phylogenetic analysis.

Sequences of Ktr proteins were downloaded from the PubMed website and used for generating the alignment using MUSCLE v3.8.31 software (www.ebi.ac.uk/Tools/msa/muscle/). The software trimAl v3 was used to trim the alignments generated by MUSCLE, with the options –gt 0.9 (for the fraction of sequences with a gap allowed) and –cons 60 (for the minimum percentage of the positions in the original alignment to conserve). ProTest v2.4 software was used to select the best protein substitution model, namely, LG+I+G+F. Finally, maximum likelihood analyses were conducted with PHYML v3.0.1 to reconstruct phylogenetic trees. Support for the branches was determined from bootstrap analysis of 100 resampled data sets.

### Nucleic acid manipulation.

Genomic DNA was extracted as previously described (46). For Southern blot analysis, 10 µg of digested genomic DNA was subjected to size fractionation on 0.7% agarose and was blotted onto a positively charged nylon membrane (Hybond-*N* +; Amersham). For DNA extraction, mycelium was grown for 16 h at 37°C in Sabouraud liquid medium. For transformation experiments, MM was used. MM was supplemented with 150 µg/ml hygromycin (Sigma) to isolate transformants. Cells of the E. coli T7 Shuffle strain, used for recombinant protein expression analysis, were grown on Luria-Bertani medium (47).

### Construction of the *Δktr* deletion and revertant strains.

A single-deletion mutant was constructed in the CEA17_Δ*akuB*^KU80^ background (44) using the β-rec/six site-specific recombination system (17). The self-excising β-rec/six blaster cassette containing the hygromycin resistance marker was released from plasmid pSK529 via the use of an FspI restriction enzyme. Using GeneArt seamless cloning and assembly (Life Technologies, Carlsbad, CA, USA), the *KTR* replacement cassettes containing the marker module flanked by 5′ and 3′ homologous regions of the target gene generated by PCR (see Table S2 in the supplemental material) were cloned into the pUC19 vector. The corresponding replacement cassettes were released from the resulting vector via the use of FspI. The CEA17_Δ*akuB*^KU80^ parental strain was transformed with the *KTR* replacement cassettes by electroporation to generate the single-deletion mutant. The transformants obtained were analyzed by diagnostic PCR and Southern blotting using the digoxigenin (DIG) probe protocol (Roche Diagnostics) (see Fig. S1 in the supplemental material). For the construction of the revertant strain, the mutant strain was cultivated in minimal medium without dextrose and with 2% xylose, which induces the excision of the selectable marker by recombination of the six recognition regions. The excision of the marker was verified by the absence of growth on malt medium containing the selectable marker. This excised strain was transformed with the *KTR* complementation cassette following the same protocol of transformation and control. All the primers used in this work are listed in Table S2.

10.1128/mBio.02647-18.8 TABLE S2 Sequence of used primers for construction of KTR mutants in A. fumigatus. Download Table S2, DOCX file, 0.03 MB. Copyright © 2019 Henry et al. 2019 Henry et al. This content is distributed under the terms of the Creative Commons Attribution 4.0 International license.

### Growth of the *Δktr* mutants.

The mycelial growth was assessed in MM and Sabouraud agar media after spotting 10³ conidia (5 µl) of each strain. Petri dishes were incubated for 48 h at 37°C and 50°C. Growth was also monitored in Sabouraud liquid media as follows: 150-ml flasks with 50 ml of media were inoculated with 10^6^ conidia and incubated with shaking (150 rpm/min) at 37°C for 24 h, and the mycelial dry weight was estimated after drying at 80°C until a constant weight was reached.

### Conidiation and conidial morphology.

Conidia (3 weeks old) were recovered from malt agar slants by the use of 0.05% Tween 20 aqueous solution. Conidial suspensions were filtered on a 40-µm-pore-size sterile cell strainer (Fisher Scientific), and conidia were counted using a Luna dual-fluorescence cell counter (Mokascience SARL). Permeability of the conidia with respect to FITC was investigated by incubating 200 µl of an aqueous suspension of conidia (2.10^7^ conidia/ml) with 30 µl of FITC solution (1 mg/ml in 0.1 M Na_2_CO_3_, pH 9) during 3 h at room temperature in darkness. The conidia were washed three times with 0.05% Tween 20 solution before being subjected to observation under a fluorescence microscope (Evosfl Life Technologies; excitation wavelength [λex], 470/22 nm; emission wavelength [λem], 510/44 nm). For calcofluor white (CFW) staining, the conidial suspension was incubated with CFW solution (0.5 µg/ml) for 30 min at room temperature and observed with fluorescence microscopy (Evos FL Life Technologies; λex, 357/44 nm; λem, 447/60 nm).

To investigate conidial survival, conidia were kept in a Tween 20 (0.05%) aqueous solution at 4°C for up to 2 months and survival over time was estimated by CFU quantification on malt agar plates. Conidial germination was followed on Sabouraud agar medium for up to 7 h at 37°C. To follow the kinetics of germination, conidia were inoculated (final suspension, 5 × 10^5^/ml) in 8-well plates (IBIDI) in liquid MM buffered with 165 mM MOPS (morpholinepropanesulfonic acid; pH 7) at 37°C. Films were recorded under a Nikon light microscope (magnification, ×40; 1 photo/4 min) and analyzed by the use of ICY software (Institut Pasteur, France). The size of conidia was estimated under a microscope using logiciel Image J software (National Institutes of Health, USA).

### Susceptibility of the *Δktr* mutant strains to antifungal compounds.

To investigate the susceptibility of mutant and parental strains to antifungal compounds, 5 × 10³ conidia were spotted on MM plates containing serial dilutions of the following compounds: CFW (2 to 30 µg/ml), Congo red (2.5 to 40 µg/ml), H_2_O_2_ (0.3 to 5 mM), and SDS (0.001% to 0.1%). Sensitivity to menadione (0.015 to 160 µM) was tested in liquid MM with resazurin method as previously described (48). Susceptibility to antifungal drugs was estimated by the Etest strips according to manufacturer recommendations (Bio-Merieux). Plates were incubated for 18 h at 37°C in a humid atmosphere. The MIC was determined as the lowest drug concentration at which the border of the elliptical inhibition zone intercepted the scale on the antifungal strip.

### Pathogenicity of the *Δktr4* mutant.

For virulence assays in the mouse model of invasive aspergillosis, immunosuppression of mice was induced with 150 mg/kg of body weight of cyclophosphamide (Pras-Farma, Barcelona, Spain) administered intraperitoneally (i.p.) and 112 mg/kg of cortisone 21-acetate (Sigma; catalog no. C-3130) administered subcutaneously, on both day −3 and day −1. Afterward, only cyclophosphamide (150 mg/kg) was used every 3 days until completion of the experiment. The body weight of each mouse was recorded weekly in order to adjust the immunosuppression dosage. Conidial inocula were prepared by growing the strains on malt–6% KCl agar slants. Conidia were harvested using saline solution containing 0.01% Tween 20 (Sigma), washed, filtered through a 40-μm-pore-size nylon sterile filter (to avoid clumping of conidia), and then counted in a hematocytometer chamber, and the concentration was then adjusted to 5 × 10^6^ conidia/ml. On day 0, the animals were anaesthetized intramuscularly with 0.1 ml of a mixture of ketamine (Ketolar; Pfizer) (50 mg/ml) and xilacina clorhidrato (Rompum; Bayer) (2%) at final concentrations of 12.5 and 2 mg/ml, respectively, and then intranasally inoculated with 30 µl of saline solution containing a total inoculum of 1.5 × 10^5^ conidia per mice. The rates of survival of mice were plotted against time, and *P* values were calculated using the log rank (Mantel-Cox) test and GraphPad Prism 5. A *P* value of <0.05 was considered signiﬁcant. Additionally, lungs removed from mice at 3 days postinfection were ﬁxed in 10% neutral buffered formalin and embedded in paraffin, and 4-micron-thick serial sections were cut and stained with hematoxylin and eosin (HE) (to enable descriptions of histopathological lesions) and Grocott’s methenamine silver (to detect fungi).

### Carbohydrate analysis of the cell wall and culture supernatant.

After 24 h of growth of the mycelium in Sabouraud liquid medium at 37°C with shaking at 150 rpm, mycelia and culture supernatants were separated by filtration. Cell wall fractions (alkali-soluble and alkali-insoluble fractions) were obtained after mycelium disruption and centrifugation as previously described (26). Polysaccharides from the cell wall were separated with respect to the function of their alkali solubility (26). Neutral hexoses were estimated by the phenol sulfuric method using glucose as the standard (49). Glucosamines (Osamines) were quantified by high-performance liquid chromatography (HPLC) after acid hydrolysis with 6 N HCl at 100°C for 6 h (50). Monosaccharides were identified and quantified by gas-liquid chromatography (GLC) after acid hydrolysis with 4 N trifluoroacetic acid at 100°C for 4 h (26).

N-Glycosylation of secreted proteins was investigated according to a previously described protocol (51). Briefly, 1 mg of protein was subjected to denaturation in 0.6 M Tris-HCl–6 M guanidium chloride for 1 h at 50°C, reduced with 20 mM dithiothreitol (DTT) for 4 h at 100°C, and finally alkylated with 110 mM iodoacetamide overnight at room temperature. After dialysis against water and freeze-drying, samples were digested with trypsin and then with PNGase F to release N-glycans. N-glycans were purified by solid-phase extraction using a C_18_-SepPak column (Waters) and paper chromatography (Whatmann no. 3) and then analyzed by MALDI-TOF.

### Matrix-assisted desorption ionization–time of flight (MALDI-TOF) mass spectrometry.

MALDI-TOF mass spectra were acquired on an UltrafleXtreme mass spectrometer (Bruker Daltonics, Bremen, Germany). Mass spectra were acquired in positive reflectron mode using FlexControl software. Mass spectra were externally calibrated in the *m*/*z* range of 700 to 3,500 Da with a peptide standard mixture (Bruker-Daltonics, Germany) and with a malto-oligosaccharide mixture. Data were analyzed with Flexanalysis software (Bruker). Samples were prepared by mixing 2 µl of an oligosaccharide solution–water (0.01 to 2 nmol)–2 µl 2 mg/ml NaCl solution with 4 µl of 2,5-dihydroxybenzoic acid matrix solution (10 mg/ml in CH_3_OH/H_2_O, 50:50 [vol/vol]). The samples (1 µl) were spotted on the target (MTP target; Bruker) and dried at room temperature.

### Production and enzymatic assay of recombinant Ktr4 protein.

The DNA sequence of *KTR4* was synthetized by the use of Geneart gene synthesis (Thermo Fisher Scientific) using an E. coli codon table optimized by the manufacturer. Furthermore, the N-terminal sequence (amino acids [aa] 1 to 28), predicted as a signal peptide cleavage site by SignalP software, was removed. The construct was cloned in the pET28a(+) expression vector using N-terminal His tagging, and the E. coli T7 Shuffle strain (New England Biolabs) was used for the protein production. The production of recombinant protein was undertaken after IPTG (isopropyl-β-d-thiogalactopyranoside) induction as follows. A 400-ml volume of LB containing kanamycin (30 µg/ml) was inoculated at an optimal density of 0.05 and shaken at 20°C and 150 rpm. When an optical density at 600 nm (OD_600_) of 0.6 was reached, induction was performed by the addition of 1 mM IPTG for 18 h. After centrifugation (3,300 × g, 10 min), the bacterial pellet was lysed with 8 mg of lysozyme–40 ml of 50 mM Tris-HCl buffer (pH 8) for 40 min at room temperature. The supernatant containing the tagged protein was purified using nickel-nitrilotriacetic acid (Ni-NTA) agarose beads (Life Technologies) according the manufacturer’s instructions. The protein was eluted with 250 mM imidazole, concentrated, and desalted using Amicon cells with a 10-kDa molecular weight cutoff (MWCO) (Merck Millipore). Determination of protein concentration was performed by the bicinchoninic acid (BCA) method, and 12% SDS-PAGE was used to verify protein purity. In spite of many attempts, it was impossible to produce a soluble recombinant form of the Ktr7 protein in E. coli, since it was exclusively found in inclusion bodies.

The mannosyltransferase activity assay was performed as previously described (52) with modifications. Briefly, 25 µg of recombinant protein was incubated in 28 μl of buffer (50 mM Tris, 75 mM KCl, 5% glycerol, 5 mM MnCl_2_, 5 mM MgCl_2_ [pH 7.5], 2 mM DTT) containing 6.7 mM GDP-Man (Sigma G5131) and 11 mM 6α-mannobiose (Dextra M206) or 2α-mannobiose (Sigma M1050) at 30°C overnight. To investigate glycoside linkages, reaction products were digested by the use of either jack bean exo-α-mannosidase (Sigma) (2.5 μl of an enzymatic reaction mixture with 1.36 U enzyme activity and 10 μl of 50 mM [pH 6.8] Na acetate buffer) or recombinant α-1,6-mannosidase (Biolabs) (2.5 μl of enzymatic reaction mixture, 80 U of α-1,6-mannosidase, 20 μl of 1× Biolabs buffer) or α-1,2-mannosidase (Prozyme) (2.5 μl of enzymatic reaction with 0.2 mU of α-1,2-mannosidase–20 μl of 1× Prozyme buffer). Mixtures were incubated for 18 h at 30°C. Digestion products were analyzed by high-pH anion-exchange chromatography (HPAEC) using a CarboPAC PA-1 column (Thermo Scientific, Villebon sur Yvette, France) (4.6 by 250 mm) and a pulsed electrochemical detector with the following gradient: an isocratic step of 98% eluent A (50 mM NaOH) and 2% eluent B (500 mM AcONa–50 mm NaOH) for 2 min, 2 to 15 min of a linear gradient (98% A/2% B to 65% A/35% B), 15 to 35 min of a linear gradient (65% A/35% B to 30% A/70% B), 35 to 37 min of a linear gradient (30% A/70% B to 0% A/100% B), 37 to 40 min under isocratic conditions with 100% B. The column was stabilized for 20 min under the initial conditions before injection was performed. Enzymatic products were purified by gel filtration chromatography on a TSK-Gel G-oligo-PW column (Tosoh Bioscience, Stuttgart, Germany) (7.8 by 300 mm) eluted with water at 0.5 ml/min. The column was calibrated with a mixture of malto-oligosaccharides. The *M*_w_ of the products was also analyzed by MALDI-TOF.

### Ethics statement.

All animal experiments performed at the Mycology Reference Laboratory were ethically approved by the Animal Welfare Committee of Instituto de Salud Carlos III and performed under the approved project license PROEX 324/16.

### Statistical analysis.

At least three biological replicates were performed per experiment; the statistical significance of the results was evaluated by a one-way variance analysis using JMP1 software (SAS Institute, Cary, NC, USA).
