# Supplementary material for: Two KTR Mannosyltransferases Are Responsible for the Biosynthesis of Cell Wall Mannans and Control Polarized Growth in Aspergillus fumigatus
Source: mBio. 2019 Feb 12;10(1):e02647-18. doi: 10.1128/mBio.02647-18 (PMC6372797; doi:10.1128/mBio.02647-18)
Supplement: FIG S1 [file mBio.02647-18-sf001.pdf]

Figure S1

A

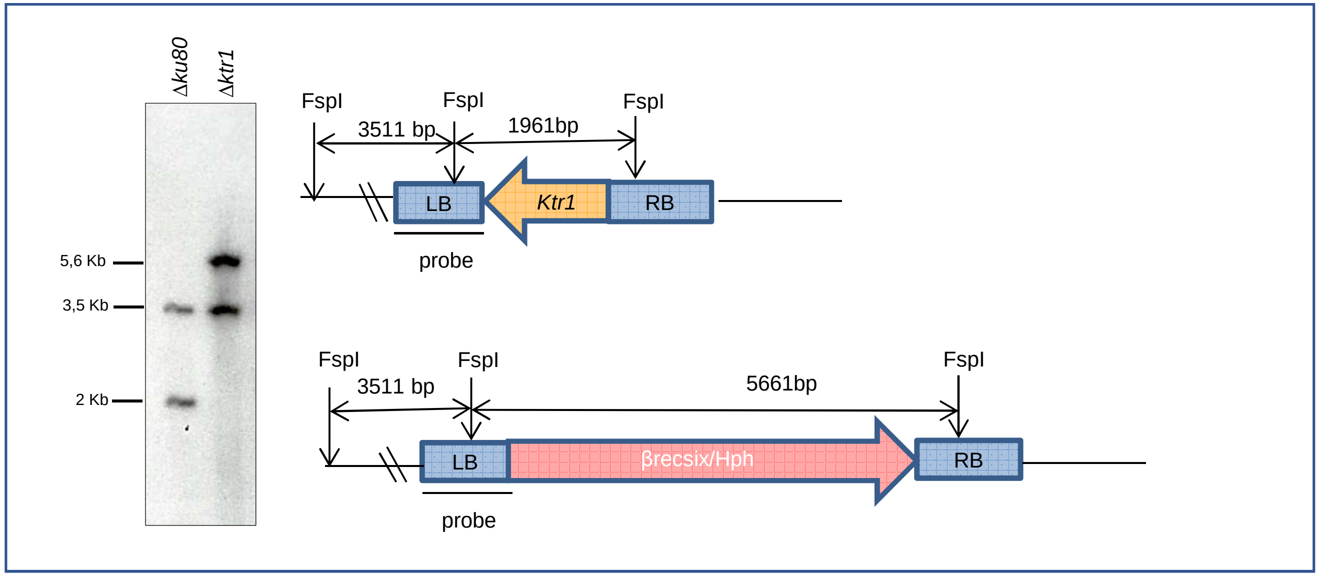

Δku80
Δktr1
5,6 Kb
3,5 Kb
2 Kb
FspI
FspI
FspI
3511 bp
1961bp
LB
Ktr1
RB
probe
FspI
FspI
FspI
3511 bp
5661bp
LB
βrecsix/Hph
RB
probe

B

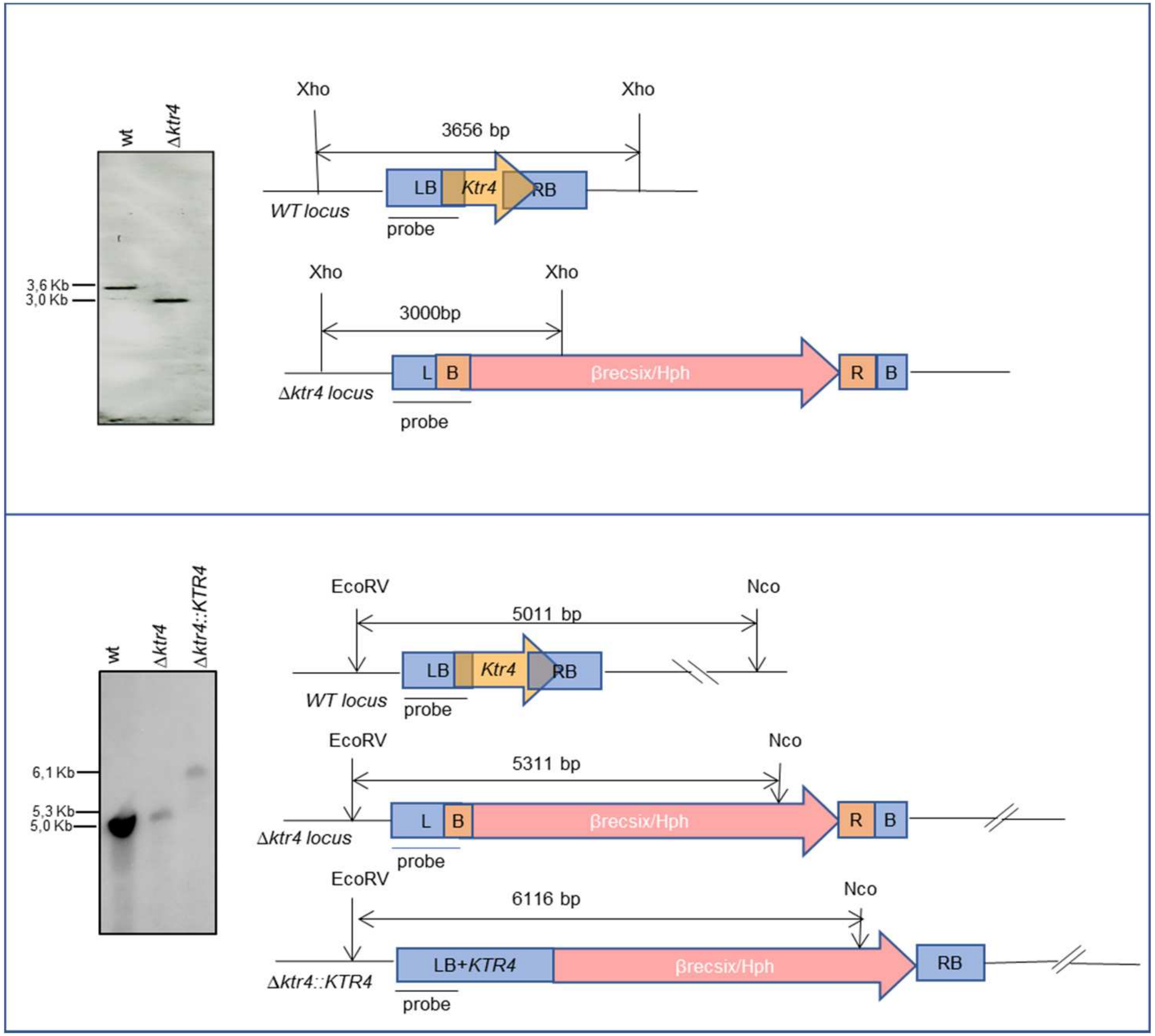

wt
Δktr4
3,6 Kb
3,0 Kb
Xho
Xho
3656 bp
LB
Ktr4
RB
WT locus
probe
Xho
Xho
3000bp
L
B
βrecsix/Hph
R
B
Δktr4 locus
probe
wt
Δktr4
Δktr4::KTR4
6,1 Kb
5,3 Kb
5,0 Kb
EcoRV
Nco
5011 bp
LB
Ktr4
RB
WT locus
probe
EcoRV
Nco
5311 bp
L
B
βrecsix/Hph
R
B
Δktr4 locus
probe
EcoRV
Nco
6116 bp
LB+KTR4
βrecsix/Hph
RB
Δktr4::KTR4
probe

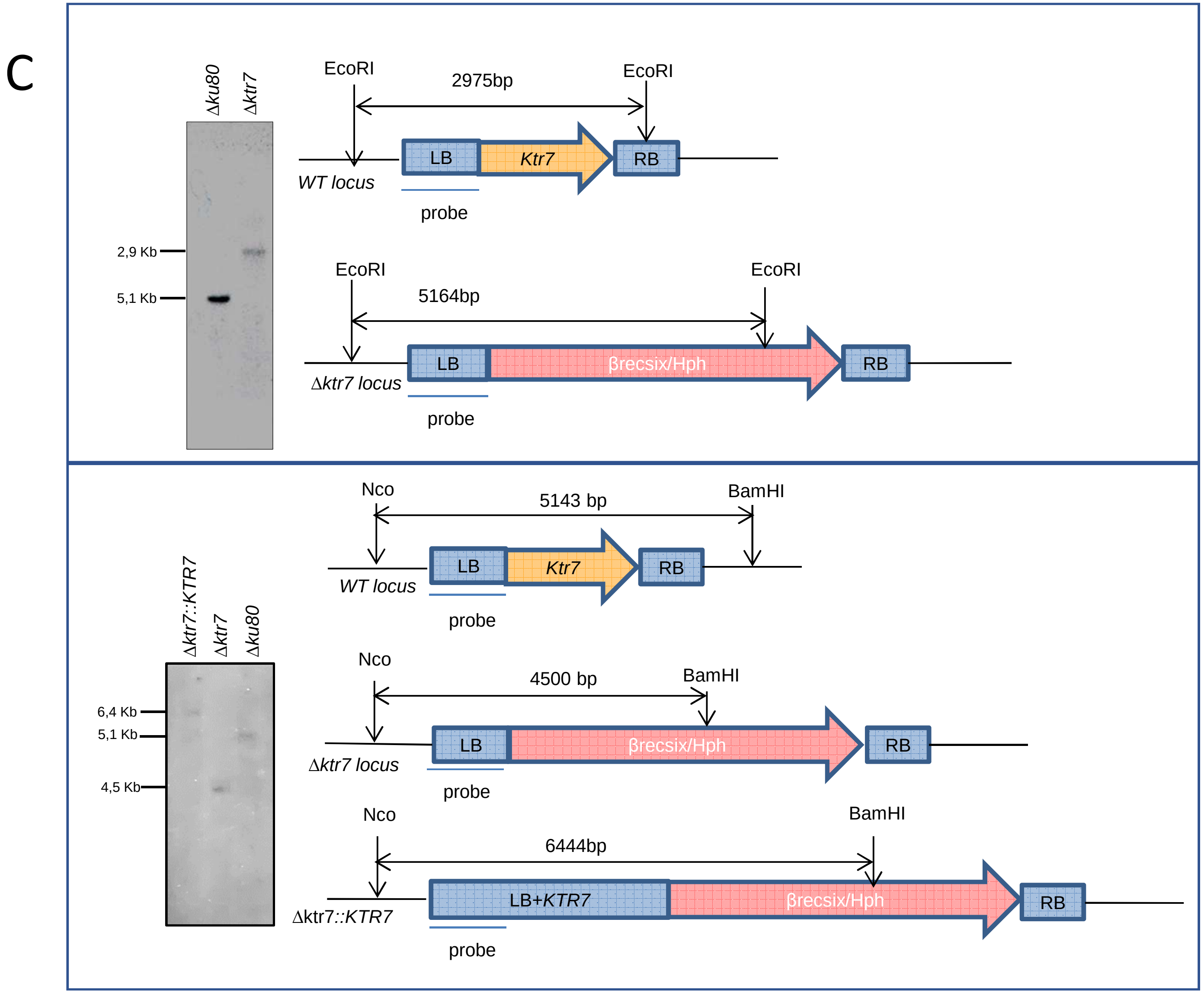

Figure S1: Southern blots of *Δktr1* (A) *Δktr4 (B)* and *Δktr7 (C)* mutants, *Δktr4::KTR4* and *Δktr7::KTR7* revertant strains and targeted replacement strategies used for *Af KTR* genes.

Each panel shows the restriction maps of the *KTR* deletion constructs after the integration of the βrec/hygromycin resistance marker at the *KTR* locus and the Southern blot analysis of the parental strain, *ktr* mutant. Genomic DNA of each strain was digested with appropriate restriction enzymes and hybridized with specific probe. Revertant strains, *Δktr4::KTR4* and *Δktr7::KTR7*, were obtained after excision of the βrec/hygromycin marker, then reintroduction of respective parental gene using the reusable βrec/hygromycin cassette.
