# Supplementary material for: Two KTR Mannosyltransferases Are Responsible for the Biosynthesis of Cell Wall Mannans and Control Polarized Growth in Aspergillus fumigatus
Source: mBio. 2019 Feb 12;10(1):e02647-18. doi: 10.1128/mBio.02647-18 (PMC6372797; doi:10.1128/mBio.02647-18)
Supplement: TABLE S1 [file mBio.02647-18-st001.docx]

**Table S1 : MIC of antifungal drugs**

| Strains | *∆ku80* | *∆ktr1* | *∆ktr4* | *∆ktr7* | *∆ktr4 ::KTR4* | *∆ktr7 ::KTR7* |
| --- | --- | --- | --- | --- | --- | --- |
|  |  |  |  |  |  |  |
| Calcofluor White (µg/ml) | 30 | 30 | **7.5** | **7.5** | 30 | 30 |
| Congo Red (µg/ml) | 40 | 40 | **2.5** | **2.5** | 40 | 40 |
| SDS (%) | 0.1 | 0.1 | **0.01** | **0.01** | 0.1 | 0.1 |
| H_2_O_2_ (mM) | 1 - 5 | 1 - 5 | 1 - 5 | 1 - 5 | 1 - 5 | 1 – 5 |
| Menadione (µM) | 20 | 20 | 20 | 20 | 20 | 20 |
| Amphotericin B (µg/ml) | 0.5 | 0.5 | 0.5 | 0.5 | 0.5 | 0.5 |
| Itraconazole (µg/ml) | 1 | 1 | 1 | 1 | 1 | 1 |
| Posaconazole (µg/ml) | 0.38-0.5 | 0.38 | 0.5 | 0.38-0.5 | 0.5 | 0.38-0.5 |
| Caspofungin (µg/ml) | 0.016 | 0.023 | 0.016 | 0.016 | 0.016 | 0.016 |
