# Supplementary material for: Two KTR Mannosyltransferases Are Responsible for the Biosynthesis of Cell Wall Mannans and Control Polarized Growth in Aspergillus fumigatus
Source: mBio. 2019 Feb 12;10(1):e02647-18. doi: 10.1128/mBio.02647-18 (PMC6372797; doi:10.1128/mBio.02647-18)
Supplement: FIG S2 [file mBio.02647-18-sf002.pdf]

Figure S2

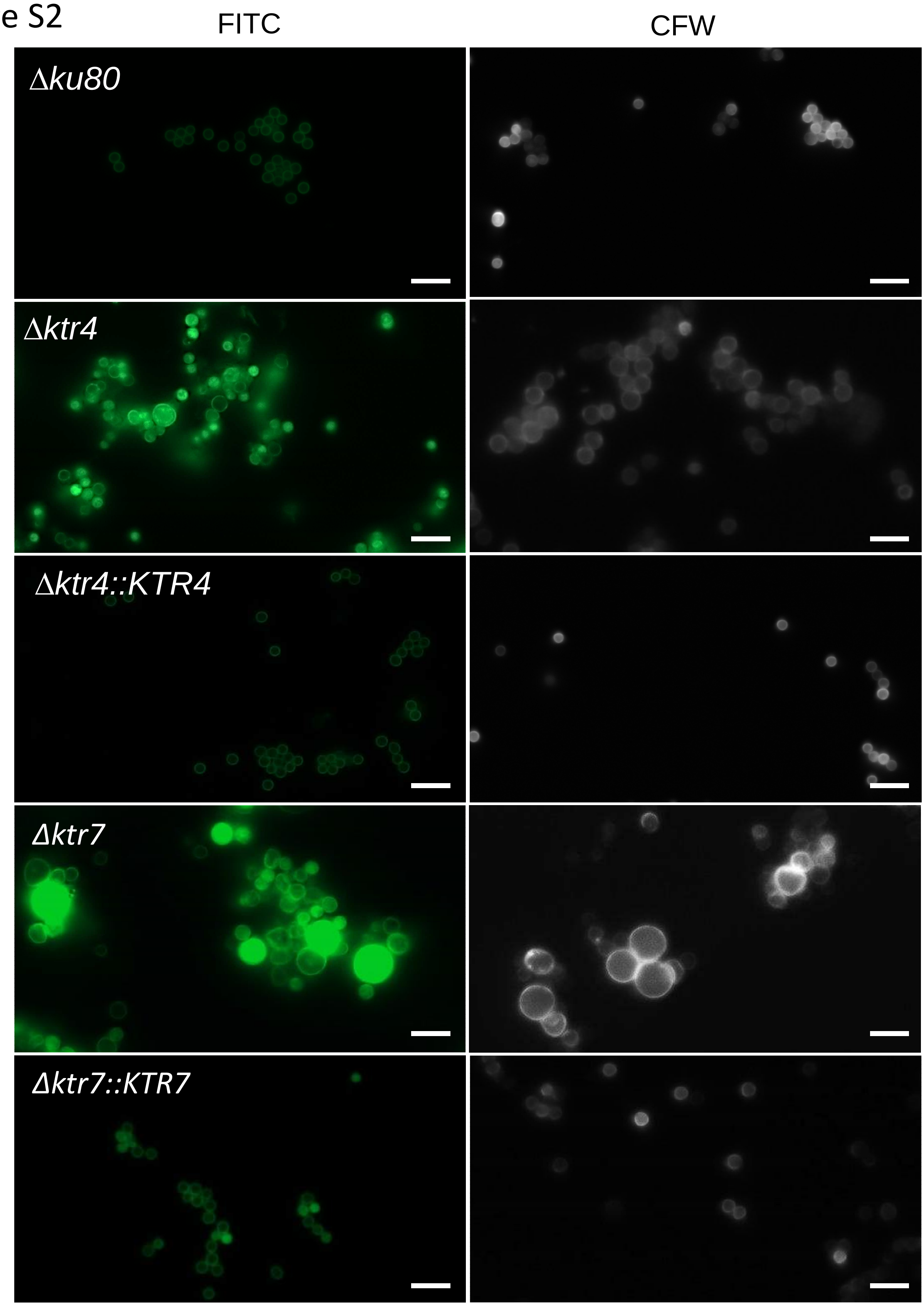

Figure S2: Conidial morphology of parental *Δku80 strain*, *Δktr4* and *Δktr7* mutants, *Δktr4::KTR4* and *Δktr7::KTR7* revertant strains.

Conidia were collected after three weeks of growth on malt/6% KCl solid medium at room temperature. Conidia were stained either with FITC (1 mg/ml) or Calcofluor white (0.5 µg/ml) and observed under fluorescent microscope. White bars represent 10 µm.
