# Supplementary material for: Two KTR Mannosyltransferases Are Responsible for the Biosynthesis of Cell Wall Mannans and Control Polarized Growth in Aspergillus fumigatus
Source: mBio. 2019 Feb 12;10(1):e02647-18. doi: 10.1128/mBio.02647-18 (PMC6372797; doi:10.1128/mBio.02647-18)
Supplement: FIG S3 [file mBio.02647-18-sf003.pdf]

Figure S3

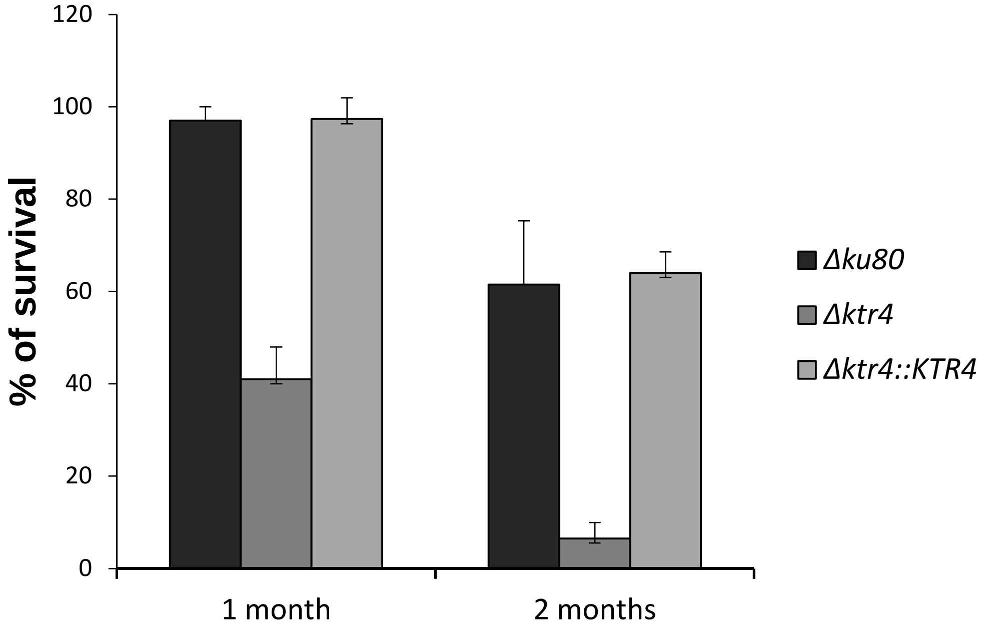

Figure S3: Conidial viability of parental *Δku80 strain*, *Δktr4* mutant and *Δktr4::KTR4* revertant strain. Conidial viability was estimated after the storage of 10^3 conidia/ml 0.05% tween 20 solution at 4°C for 1 and 2 months. The percentage of survival was estimated by CFU after spreading on a 2% malt agar plates. Each value was the average of three independent replicates (Error bars represent standard deviation).
