# Supplementary material for: Two KTR Mannosyltransferases Are Responsible for the Biosynthesis of Cell Wall Mannans and Control Polarized Growth in Aspergillus fumigatus
Source: mBio. 2019 Feb 12;10(1):e02647-18. doi: 10.1128/mBio.02647-18 (PMC6372797; doi:10.1128/mBio.02647-18)
Supplement: FIG S4 [file mBio.02647-18-sf004.pdf]

Figure S4

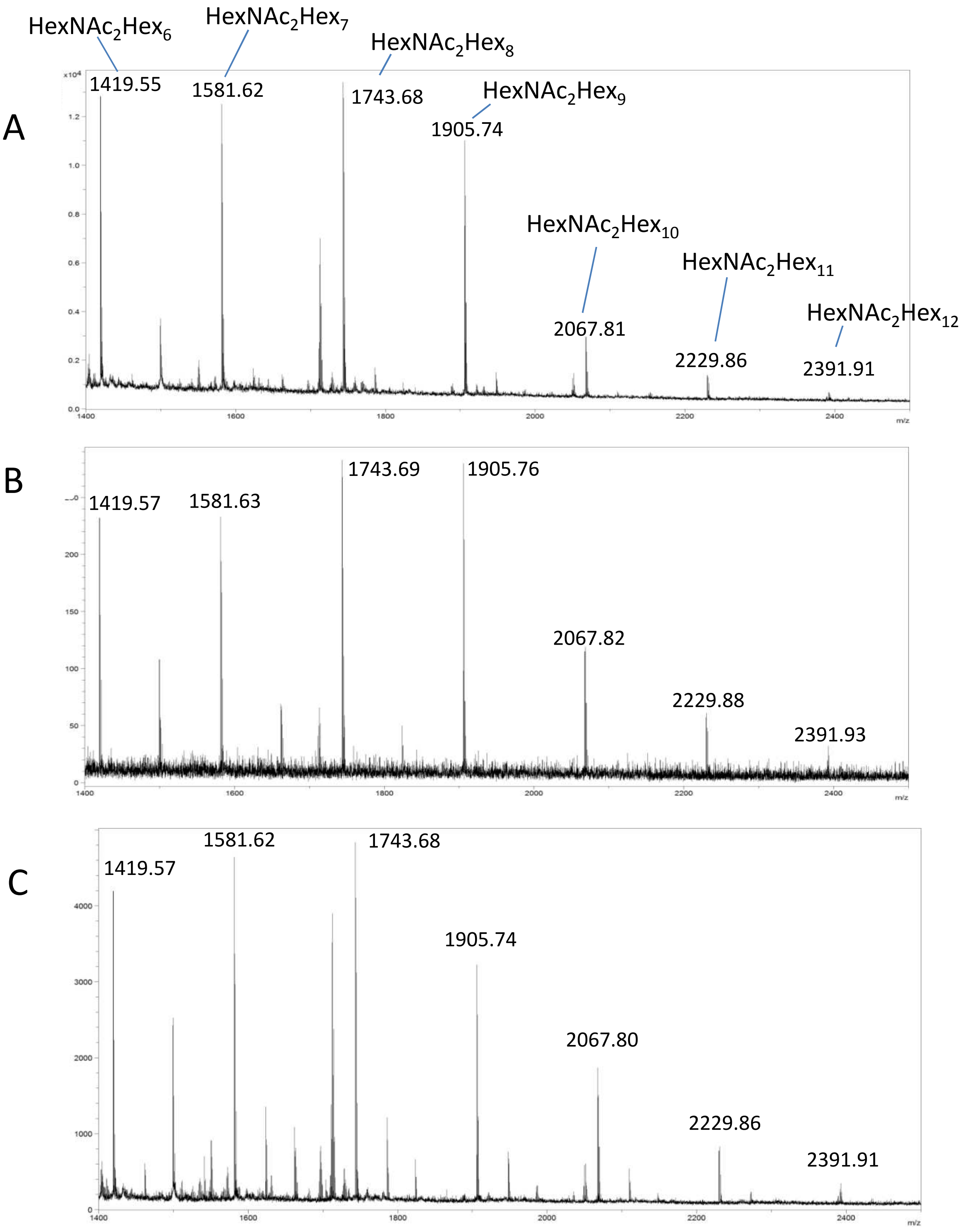

Figure S4: MALDI-TOF mass spectra of N-glycans

MALDI-TOF mass spectra of N-glycans purified from secreted proteins produced by parental *Δku80* strain(A), *Δktr4* (B) and *Δktr7* (C) mutants in liquid Sabouraud medium. Mass spectra were acquired using Flexcontrol software and shots were recorded in positive reflectron mode. Ion mass (m/z) correspond to $[M + Na]^+$ (HexNac, N-Acetylhexosamine; Hex, Hexose)
