# Supplementary material for: Two KTR Mannosyltransferases Are Responsible for the Biosynthesis of Cell Wall Mannans and Control Polarized Growth in Aspergillus fumigatus
Source: mBio. 2019 Feb 12;10(1):e02647-18. doi: 10.1128/mBio.02647-18 (PMC6372797; doi:10.1128/mBio.02647-18)
Supplement: FIG S5 [file mBio.02647-18-sf005.pdf]

Figure S5

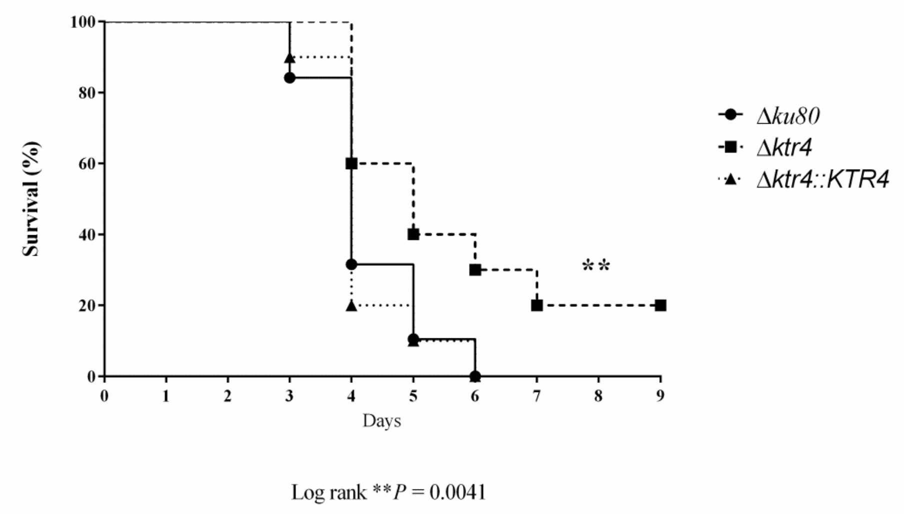

Figure S5: Virulence of the parental *Δku80*, *Δktr4* mutant and *Δktr4::KTR4* revertant strains in invasive aspergillosis mouse model.
