# Supplementary material for: Two KTR Mannosyltransferases Are Responsible for the Biosynthesis of Cell Wall Mannans and Control Polarized Growth in Aspergillus fumigatus
Source: mBio. 2019 Feb 12;10(1):e02647-18. doi: 10.1128/mBio.02647-18 (PMC6372797; doi:10.1128/mBio.02647-18)
Supplement: FIG S6 [file mBio.02647-18-sf006.pdf]

Figure S6

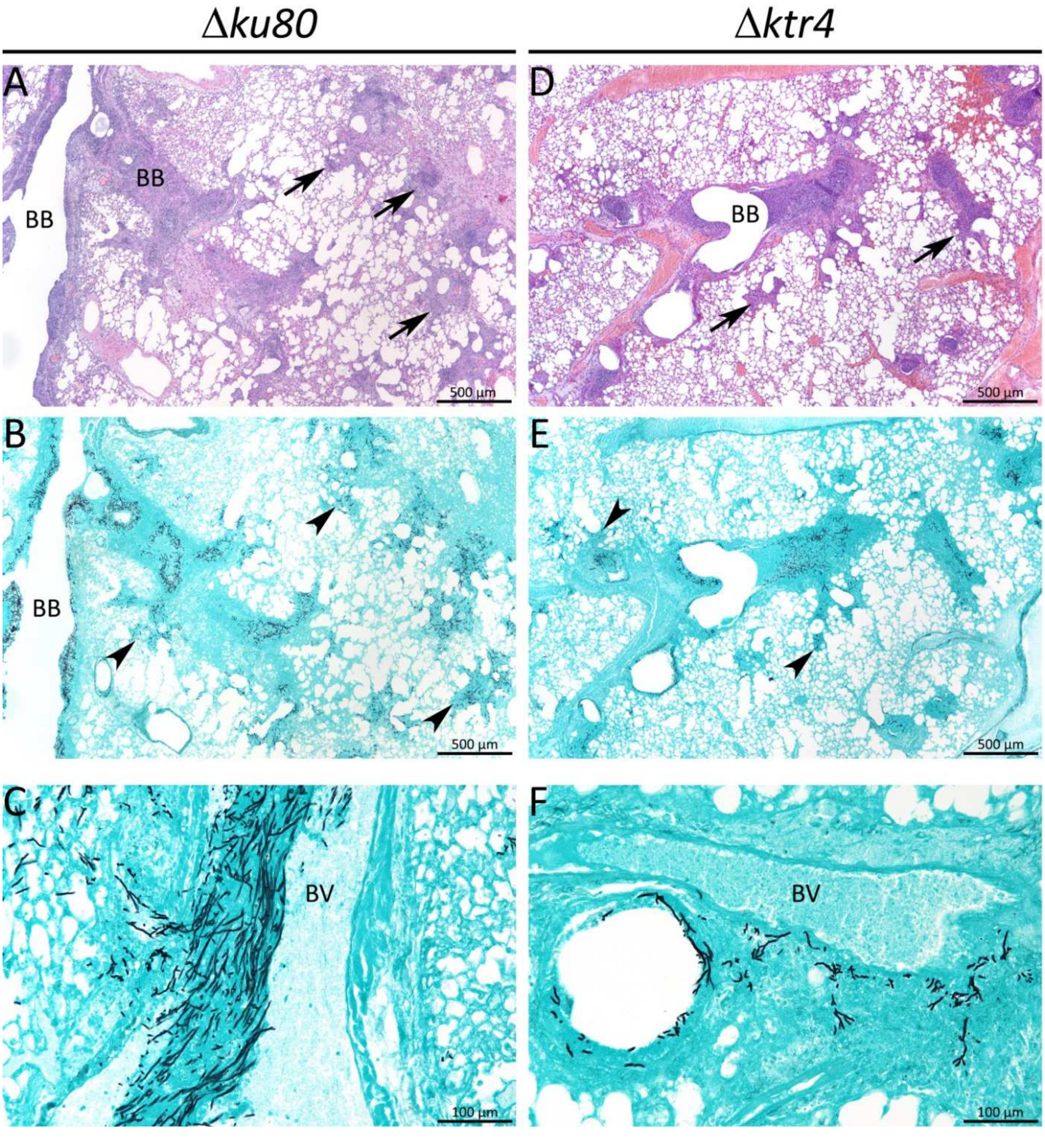

Figure S6: Histopathological sections of mouse lungs infected with *A. fumigatus* strains:
Panels A-C, parental strain (*Δku80*); Panels D-F, *Δktr4* mutant
(Panels A and D: HE staining; Panels B, C, E and F: Gomori Grocott's staining).
***Lung section with Δku80 strain* (Panels A-C):** Multifocal inflammatory lesion, centered on bronchi/bronchioles (BB), with secondary extension to alveoli (black arrows), containing filamentous fungi (black arrow heads)(**A, B**). Fungi were located in the bronchi/bronchioles, with invasion of alveoli (black arrowheads, **B**) and large blood vessels (BV; **C**).
***Lung section with Δktr4* mutant (D-F):** Same inflammatory lesion as *Δku80* (**D, E**), with less invasion of alveoli by the fungus (black arrowheads, **E**) and no invasion of large blood vessels (**F**).
