# Supplementary material for: Two KTR Mannosyltransferases Are Responsible for the Biosynthesis of Cell Wall Mannans and Control Polarized Growth in Aspergillus fumigatus
Source: mBio. 2019 Feb 12;10(1):e02647-18. doi: 10.1128/mBio.02647-18 (PMC6372797; doi:10.1128/mBio.02647-18)
Supplement: TABLE S2 [file mBio.02647-18-st002.docx]

**Table S2 : Sequence of used primers for construction of KTR mutants in *A. fumigatus***

| **Primers** | **Sequence** |
| --- | --- |
| FG ktr1 FW | AATTCGAGCTCGGTACAAGCTTAAAGGCATCTTGATCGTCGT |
| FG ktr1 RV | GGACCTGAGTGATGCGCTCCTGGTGAGCAAAATGT |
| FD ktr1 FW | TGGTCCATCTAGTGCTTCGATGCTCTTGACCACGA |
| FD ktr1 RV | GCCAAGCTTGCATGCCAAGCTTCAACTGTCCATTGCGCTAGA |
| ctrl Ktr1 FW | CTTCTCTAGGCCTGATCGCAAT |
| ctrl Ktr1 RV | CGGGCAATGTGTGAATGGAA |
| FG ktr7 FW | AATTCGAGCTCGGTACAAGCTTTGCACTGTGTACTCCCTTGT |
| FG ktr7 RV | GGACCTGAGTGAAATGTCAGGTTCCAGCGGTA |
| FD ktr7 FW | TGGTCCATCTAGCTGATTTGCCTCTGGAACCC |
| FD kre2.2 RV | GCCAAGCTTGCATGCCAAGCTTCAATGACGCATATGGAGGGC |
| ctrl ktr7 FW | TCCTGTCCATATTTCGGCGA |
| ctrl Ktr7 RV | AGCACGCTAGATGAACCTGT |
| ext FG Ktr7 | AGCACACGCACTCACAAAAG |
| ext FD Ktr7 | AACATGATGCCATCCTCTCC |
| FG ktr4 FW | AATTCGAGCTCGGTACTGCGCACTCCCCGAGTGTTCCCCATC |
| FG ktr4 RV | GGACCTGAGTGATGCGGTCCCAGTGTTCCTTTGGC |
| FD ktr4 FW | TGGTCCATCTAGTGCTGGGATGGTTGACGGACGAC |
| FD ktr4 RV | GCCAAGCTTGCATGCCTGCGCAGGTGAATGGAGGCGCACATG |
| ext FG ktr4 | CAGGCCCTCTAGCTGTTTTG |
| ext FD ktr4 | GTCTCAACGACTTCCCCACT |
| ctrl ktr4 FW | GCCAGAGGAGGGAAGAAGTT |
| ctrl ktr4 RV | AGTGAAGACCCGAACTCCTG |
| FG cpl ktr7 FW | AATTCGAGCTCGGTACTCTAGATGCACTGTGTACTCCCTTGT |
| FG cpl ktr7 stop RV | GGACCTGAGTGACTATGGAGATGCCCATCCTC |
| FD cpl ktr7 FW | TGGTCCATCTAGCTGATTTGCCTCTGGAACCC |
| FD cpl ktr7 RV | GCCAAGCTTGCATGCCTCTAGACAATGACGCATATGGAGGGC |
| FG cpl Ktr4 FW | AATTCGAGCTCGGTACTGCGCACTCCCCGAGTGTTCCCCATC |
| FG cpl Ktr4 stop RV | GGACCTGAGTGATGCTTAATGCATCCCCACATGCT |
| FD cpl Ktr4 FW | TGGTCCATCTAGTGCCCCATGAAATTGTTTCTTAATTCC |
| FD cpl Ktr4 RV | GCCAAGCTTGCATGCCTGCGCACAAGAACCCCGTTTCTTTGT |
